# Psychosomatic treatment for allergic diseases

**DOI:** 10.1186/s13030-015-0036-2

**Published:** 2015-03-18

**Authors:** Kazufumi Yoshihara

**Affiliations:** Department of Psychosomatic Medicine, Graduate School of Medical Sciences, Kyushu University, 3-1-1 Maidashi, Higashiku, Fukuoka 812-8582 Japan

**Keywords:** Allergy, Stress, Asthma, Psychosomatic assessment, Psychosomatic treatment, Psychosomatic disorder, Guideline

## Abstract

Many reports have been published concerning how psychosocial stress influences the occurrence and progression of allergic diseases such as bronchial asthma and atopic dermatitis. As for asthma, a typical allergic disease often accompanied by psychosomatic related problems, the Global Initiative for Asthma (GINA), international medical guidelines for asthma, describes psychosocial problems as causative factors of poor asthma control and as risk factors for asthma exacerbation, even if symptoms are well controlled. However, because there is little high quality evidence for effective treatments for asthma patients with psychosocial problems, concrete assessments and treatments for such problems is scarcely described in GINA. Therefore, psychosomatic intervention for asthma patients is not effectively conducted on a worldwide scale. In contrast, the “Japanese Guidelines for the Diagnosis and Treatment of Psychosomatic Diseases” describe the assessment and treatment of psychosomatic disorders in detail. In the guidelines, psychosocial factors are classified into five categories; 1) Relation between stress and asthma occurrence or progression, 2) Relation between emotion and asthma symptoms, 3) Problems related to a patient’s character and behaviors, 4) Problems of daily life and Quality of Life (QOL), and 5) Problems related to family relationships and life history. The employment of a self-administered questionnaire, the “Psychosomatic Questionnaire related to Asthmatic Occurrence and Progression”, is useful for clarifying psychosocial factors and for setting up treatment strategies according to the problems identified. The Japanese guidelines have been proven to be useful, but empirical evidence for their effectiveness is still relatively limited. It will be necessary in the future to accumulate high-quality evidence and to revise the psychosomatic approaches in the guidelines that are universally valid.

## Introduction

Psychosocial stress affects the nervous, endocrine, and immunological systems, which are involved in the onset and exacerbation of various diseases. Many studies have reported psychosocial influences on the onset and progression of allergic diseases such as bronchial asthma and atopic dermatitis [1-11] with psychosomatic pathology, which is defined as “pathophysiological states of somatic disorders that have been closely affected by psychosocial factors in their occurrence and progression and in which organic and/or dysfunctional lesions are found” [12]. It is important to first assess these psychosocial factors and to customize a treatment strategy by clarifying the psychosocial problems of each patient.

Concerning asthma, a typical allergic disease often accompanied by psychosomatic problems, some “preparation factors” are inherent to the pathogenesis, such as atopic disposition and airway hyperresponsiveness, while others are acquired preclinical factors (allergens, air pollutants, psychosocial stress in childhood, character, and behavioral problems). These inherent and acquired factors are together called “preparatory condition” [13], and asthma develops when an inciting factor, such as exposure to an allergen, catching a cold, or psychosocial stress, is combined. Manifested asthma may persist or worsen by various individual and environmental factors, including emotional states, personal characteristics, and behavioral problems, as well as daily life problems caused by the disease. Asthma symptoms can be improved or cured by reducing the load of such psychosocial factors.

In the Global Initiative for Asthma (GINA), international medical guidelines for asthma, it is stated that psychosocial factors are important in the control and management of asthma [14] and that psychosocial problems can be causative of poor control and exacerbations of asthma, even if symptoms are well controlled. However, how psychosocial problems should be assessed and treated is barely described. The guidelines contain descriptions such as “Drug treatment and cognitive behavior therapy have been described as having some potential in patients with asthma”, “Relaxation strategies and breathing techniques may be helpful”, and “Psychological interventions may be helpful in patients with severe asthma”. The lack of detailed descriptions implies the fact that psychosomatic treatment for asthma patients has not yet been effectively achieved in most countries.

To the contrary, the “Japanese Guidelines for the Diagnosis and Treatment of Psychosomatic Diseases” enable the physician to assess the psychosocial factors of asthma patients by use of a self-administered questionnaire specifically designed for patients with asthma: the “Psychosomatic Questionnaire Related to Asthma Occurrence and Progression” [15,16]. It is included in the guidelines, and the results can be used to set up a treatment strategy based on the answers to the questionnaire. Moreover, in the guidelines psychosomatic treatments for specific psychosocial factors are comprehensibly described in such a way that any primary physician can easily diagnose and effectively treat asthma patients with psychosomatic problems. In this review, the “Japanese Guidelines for the Diagnosis and Treatment of Psychosomatic Diseases” and recent research papers are utilized to outline the procedures for the assessment and treatment for asthma patients with psychosomatic problems.

## Psychosomatic assessment

### Basic grouping of the psychosocial factors related to psychosomatic disorders

Psychosocial factors involved in psychosomatic disorders can be classified into three categories: Preparation, inciting, and continuing and precipitating factors [17].

#### Preparation factors

Preparation factors do not directly cause the disease, but they produce preclinical conditions in which the disease occurs when inciting factors are added. They include problems of life history, family relationships, character, and behavioral styles.

#### Inciting factors

Inciting factors are acquired ones that manifest the disease in combination with one or more of the above listed preparation factors. Intense emotional stressors that cause fear, anger, or sadness are examples of inciting factors.

#### Continuing and precipitating factors

Continuing factors or precipitating factors are also acquired ones that prolong or worsen the course of the disease. Psychosocial factors that can be continuing and precipitating factors include problems of emotion, character, behavior, and daily life caused by the disease.

### The classification and assessment of psychosocial factors related to asthma

In the section “bronchial asthma” of the “Japanese Guidelines for the Diagnosis and Treatment of Psychosomatic Diseases”, procedures for assessing and clarifying problems related to the psychosocial background of the patient and strategies of treatment adjusted to those problems are given. Psychosocial problems are classified into the following five groups.

#### Relation between stress and asthma onset and progression

Psychosocial stressors have been reported as inciting, continuing, or precipitating factors for asthma [4-11]. Many of them are related to major life events (entering school, being employed, having a child, divorce, moving, changing jobs, and a close relative’s sickness or death) or to minor stressors in everyday life (problems with interpersonal relationships in family, at school, or at workplace and a heavy burden of study or work). These psychosocial stressors are often associated with the onset, continuation, exacerbation, or recurrence of asthma symptoms. Possible mechanisms linking stressors and asthma onset or exacerbations are the production of cytokines, which can be modified by stressors and heightened susceptibility to infection related to deterioration of the immune system through stress [18,19].

#### Relation between emotion and asthma symptoms

A close relation has been shown between emotion (anxiety, tension, anger, depression, etc.) and the exacerbation or remission of asthma symptoms [20-25]. A high rate of panic disorder has been reported for asthmatics, which can worsen asthmatic symptoms [22,23]. A possible mechanism is distal airway closure induced by parasympathetic nervous activation and the consequent histamine release. Depression has been reported to be a risk factor for the death of asthmatic subjects [21,24].

#### Patient character and behavioral problems

Most asthmatic patients who are scrupulous or perfectionistic and who suppress their feelings or conform to others’ opinions tend to have severe symptoms because they become fatigued by not resting even if they are tired, they cannot refuse when asked to do things, they cannot say what they want to say, they cannot consult others about the things that worry them, and they cannot seek help from people around them [10].

#### Problems of daily life and Quality of life (QOL)

Asthma patients who have repeated fluctuations of symptoms tend to have little prospect for improvement. In addition, they have great physical, mental, and economic burdens related to their asthma symptoms and treatment. These burdens can cause remarkable psychological distress and social or occupational dysfunction, as well as sleep disturbance, difficulties in interpersonal relationships, social withdrawal, decreased achievement of study or work, depressive mood, and anxiety.

#### Problems related to family relationships and life history

Some people who have had problems in their relationship with their parents since childhood or have experienced bullying at school may have a distrust of other persons, a sense of anxiety, and/or self-denial. As they grow up, they often retain strong conflicts in interpersonal relationships and feel anxious or stressful when they confront such issues. Therefore, most asthma patients who have problems in their family relationships and life history have poor asthma control.

Chida et al. demonstrated in an animal study that psychological stress in childhood causes airway inflammation and worsens the hyperresponsiveness of adult-onset asthma and reported a relation between symptom exacerbation and dysfunction of the hypothalamic-pituitary-adrenal axis [26].

### Psychosomatic questionnaire related to asthma occurrence and progression

The “Psychosomatic Questionnaire related to Asthmatic Occurrence and Progression” in the “Japanese Guidelines for the Diagnosis and Treatment of Psychosomatic Diseases” is a helpful tool for physicians when they question asthma patients about their psychosocial background in a medical interview. This questionnaire is useful for getting the whole picture of the psychosocial background of asthmatic patients and for judging if the asthma patient has a psychosomatic disorder. The questionnaire consists of 25 items classified into five subcategories; 1) Relation between stress and the occurrence and progression of asthma, 2) Relation between emotion and asthma symptoms, 3) Patient character and behavioral problems, 4) Problems of daily life and QOL, and 5) Family relationship and life history problems. Patient problems are indicated by high subcategory scores, which makes it possible to understand each patient’s problems to some degree and to implement an appropriate psychosomatic treatment regimen to deal with these problems.

## Psychosomatic treatment

### Basis of psychosomatic treatment

Ago systematized psychosomatic treatment into five stages so that it is well organized and one can easily comprehend it [27]. The five stages are as follows.

## First stage: achievement of a therapeutic relationship with mutual trust and motivation to treatment

Some psychosomatic patients refuse to admit that they need psychological intervention. In such cases, first of all, somatic symptoms should be treated. Intensive physical care facilitates the establishment of mutual trust between the patient and doctor. In parallel with the physical care, detailed information about the medical history related to the occurrence and progression of the disease should be collected, from both the physical and psychological aspects. Through these interventions some patients realize the importance of mind-body correlations and are motivated to accept to the psychosomatic treatment.

## Second stage: relaxation and experiencing a reduction or the disappearance of symptoms

Patients placed in a stressful environment need environmental regulation. Although it is often difficult, separation of the patient from his/her stressful environment is desirable to help him/her attain mental and physical relaxation. Autogenic training is also effective as a relaxation technique. In addition, it is important to give the patient opportunities to express his or her repressed frustrations and negative feelings during interview sessions. The doctor’s sympathetic understanding of the patient’s problems and feelings helps the patient release his or her repressed emotion. In the case of sleep disturbance, anxiety, depression or somatic complaints, medication should be considered. When patients experience the improvement or disappearance of symptoms and have no relapse, they often have a strong desire for psychosomatic treatment.

## Third stage: understanding the mind-body correlation and making appropriate adjustments

In this stage, the approach is focused on external factors that can worsen symptoms and on the patient’s patterns of thinking and behavior. The patients become aware of their inappropriate patterns from a third person perspective. Cognitive-behavioral therapy and transactional analysis are effective.

## Fourth stage: acquisition of a more appropriate adjustment reaction

It is important to support the patients in correcting their patterns of thinking and behavior that are related to the occurrence or exacerbation of their symptoms and to assist them in acquiring appropriate new patterns. From this they will benefit by learning to regard events objectively, forgive themselves and others, and maintain proper assertions.

## Fifth stage: gradual dissolution of the therapeutic relationship

When the patients learn more appropriate styles of thinking and behavior and it is confirmed that the symptoms neither relapse nor deteriorate even if medication is tapered off and discontinued, the psychosomatic treatment is completed.

### Psychosomatic treatment for asthma

In the Japanese guidelines, psychosomatic treatment for asthma consists of approaches to problems related to asthma, such as education concerning asthma and lifestyle guidance, Comprehensive treatment I, and approaches to individual problems such as stress, character, and behavioral problems, Comprehensive treatments II and III. The details are as follows.

#### Comprehensive treatment I

The knowledge necessary to control asthma is described plainly and properly in such a way that the patient can easily understand. Patients are educated about their pathophysiology, their medication (its contents, desired effects, and adverse effects), and the precipitating factors (overwork, stress, medicine, smoking, drinking, etc.) of asthma, the management of asthma attacks, avoidance of allergens, and good lifestyle and habits. In addition, as previously mentioned, the doctor engages in the first stage of psychosomatic treatment; achieving a therapeutic relationship with mutual trust and motivation to treatment. In addition, the “Psychosomatic Questionnaire related to Asthma Occurrence and Progression” is administered.

#### Comprehensive treatment II

If asthma symptoms do not improve, it should first be determined if the patient has adhered to the treatment and if the patient has made an effort to reduce the precipitating factors. The doctor should carefully interview the patient about the causes of his/her non-compliant behaviors, then educate the patient about the importance of a constructive attitude toward the treatment.

If environmental causes of asthma exacerbation are found in the family or the workplace, counseling with family members or superiors should be held. In the case of character or behavioral problems [28], the patient should be advised about concrete ways of achieving good adherence. Moreover, when the patient has anxiety or depression, medication should be combined with psychotherapy.

#### Comprehensive treatment III

If the patient’s adherence to the treatment is not sufficient, psychotherapy to reduce the patient’s stress should be considered. High scores on subscales of the “Psychosomatic Questionnaire related to Asthma Occurrence and Progression” indicate the necessity to clarify the patient’s problems and to approach them with specific methods for each subcategory, as follows.

1. When the score for “Relation between stress and asthma occurrence or progression” is high
  As patients become aware of the mind-body correlation, they begin to accept the psychosomatic treatment without difficulty. If the patient is overworked, has insomnia, or leads an unhealthy life when his/her asthma symptoms are developed or exacerbated, the causes of those problems should be determined and resolved.
  Concerning environmental adjustments in the family or the workplace, counseling with family members or superiors at their workplace is necessary to reduce the patient’s burdens. If asthma symptoms cannot be controlled in an outpatient setting, a hospitalization with the aim of taking the patient out of stressful situations may be required. Hospital admissions often bring the patient a significant decrease in symptoms and can be a beneficial experience that helps the patient recognize mind-body correlations and gain motivation to engage in a better lifestyle.
2. When the score for “Relation between emotion and asthma symptoms” is high
  Intense emotions are precipitating factors of asthma [29]. When the patient has anxiety or depression, medication and psychotherapy are conducted simultaneously [30-33]. The symptoms of asthma may continue or worsen according to the patient’s ability to suppress negative emotions such as anger, dissatisfaction, and sadness. In such cases, the expression of suppressed emotions is essential. Moreover, the patients need to review how they cope with events that cause negative emotions.
3. When the score for “Patient’s character and behavioral problems” is high
  The symptoms of asthma may worsen when the patient overworks, has stressful interpersonal relationships, or has problems coping with stress. In such cases, the patient’s problems and their background should be clarified to help the patient recognize and modify his/her problematic behaviors. Moreover, experience in reducing symptoms through better coping with stress leads to better modification of behaviors.
  Some patients with alexithymia [34] (inability to recognize or to express emotion) cannot engage in appropriate coping strategies because they are not aware of their condition when they are confronted with a stressful situation. They need to be encouraged to identify their feelings and to express their emotions. When patients have difficulty verbally expressing or describing their feelings, sand-play or art therapy may be effective.
  Some patients with alexisomia [35] (lacking in awareness of somatic sensations) have difficulty perceiving bodily states, such as shortness of breath, and they are likely to consult a physician only after their asthma symptoms worsen. In this case, continuous monitoring of symptoms with a peak flow meter, which provides an objective measure of lung function, is helpful in training the patients to understand their physical condition.
4. When the score for “Problems of daily life and Quality of life” is high
  It has been reported that the worse the symptoms of asthma, the lower the QOL of the patient. Therefore, it is necessary to treat asthma patients from the perspective of both mind and body to improve their QOL. Asthma symptoms disturb patients’ activities at home and at their workplace. Disturbances in everyday life can induce a vicious cycle between symptoms and stress. Patients should be instructed to keep in mind that they need unprejudiced cognition and timely relaxation in daily life and not to overwork, even if they are afraid that their absence may inconvenience their family members or coworkers.
5. When the score for “Problems of family relationships and life history” is high
  One’s life history is associated with his/her character formation, ability to trust, cognition, and behavioral patterns. A detrimental life history contributes to the distortion of character, cognitive, and behavioral patterns, which causes undeveloped and stressful coping. If the patient’s life history is related to the exacerbation of asthma symptoms, the same approaches as those for the above-mentioned character and behavioral problems are chosen. If there are any problems in the patient’s family, the patient should be temporarily separated, through a hospitalization for example, from the stressful situation. When the patient has problems with an interpersonal relationship and does not respond to individual therapy, family therapy or interviews including family members may be effective.

## Conclusions

When psychosocial factors influence the occurrence or progression of allergic disease, psychosomatic treatment is necessary. Assessment of psychosocial factors and psychosomatic treatment that follow the “Japanese Guidelines for the Diagnosis and Treatment of Psychosomatic Diseases” is useful in the usual medical setting, although there is relatively limited empirical evidence at the present time. It will be necessary to revise the guidelines for the psychosomatic treatment of patients with allergic diseases by accumulating high-quality evidence and to publish it worldwide.

## Abbreviations

GINA: Global Initiative for Asthma
QOL: Quality of Life
